# Nutlin-3 Loaded Ethosomes and Transethosomes to Prevent UV-Associated Skin Damage

**DOI:** 10.3390/life14010155

**Published:** 2024-01-21

**Authors:** Elisabetta Esposito, Francesca Ferrara, Markus Drechsler, Olga Bortolini, Daniele Ragno, Sofia Toldo, Agnese Bondi, Alessandra Pecorelli, Rebecca Voltan, Paola Secchiero, Giorgio Zauli, Giuseppe Valacchi

**Affiliations:** 1 Department of Chemical, Pharmaceutical and Agricultural Sciences, University of Ferrara, I-44121 Ferrara, Italy; frrfnc3@unife.it (F.F.); daniele.ragno@unife.it (D.R.); agnese.bondi@unife.it (A.B.); 2 Bavarian Polymer Institute (BPI) Keylab “Electron and Optical Microscopy”, University of Bayreuth, D-95440 Bayreuth, Germany; mardre@mardre.com; 3 Department of Environmental Sciences and Prevention, University of Ferrara, I-44121 Ferrara, Italy; olga.bortolini@unife.it (O.B.); sofia.toldo@unife.it (S.T.); alessandra.pecorelli@unife.it (A.P.); rebecca.voltan@unife.it (R.V.); 4 Department of Translational Medicine and LTTA Centre, University of Ferrara, I-44121 Ferrara, Italy; paola.secchiero@unife.it; 5 Research Department, King Khaled Eye Specialist Hospital, Riyadh 11462, Saudi Arabia; giorgio.zauli@unife.it; 6 Plants for Human Health Institute, Animal Sciences Department, NC Research Campus, NC State University, Kannapolis, NC 28081, USA

**Keywords:** nutlin-3, ethosomes, transethosomes, skin damage, UV radiations

## Abstract

The skin’s protective mechanisms, in some cases, are not able to counteract the destructive effects induced by UV radiations, resulting in dermatological diseases, as well as skin aging. Nutlin-3, a potent drug with antiproliferative activity in keratinocytes, can block UV-induced apoptosis by activation of p53. In the present investigation, ethosomes and transethosomes were designed as delivery systems for nutlin-3, with the aim to protect the skin against UV damage. Vesicle size distribution was evaluated by photon correlation spectroscopy and morphology was investigated by cryogenic transmission electron microscopy, while nutlin-3 entrapment capacity was evaluated by ultrafiltration and HPLC. The in vitro diffusion kinetic of nutlin-3 from ethosomes and transethosomes was studied by Franz cell. Moreover, the efficiency of ethosomes and transethosomes in delivering nutlin-3 and its protective role were evaluated in ex vivo skin explants exposed to UV radiations. The results indicate that ethosomes and transethosomes efficaciously entrapped nutlin-3 (0.3% *w*/*w*). The ethosome vesicles were spherical and oligolamellar, with a 224 nm mean diameter, while in transethosome the presence of polysorbate 80 resulted in unilamellar vesicles with a 146 nm mean diameter. The fastest nutlin-3 kinetic was detected in the case of transethosomes, with permeability coefficients 7.4-fold higher, with respect to ethosomes and diffusion values 250-fold higher, with respect to the drug in solution. Ex vivo data suggest a better efficacy of transethosomes to promote nutlin-3 delivery within the skin, with respect to ethosomes. Indeed, nutlin-3 loaded transethosomes could prevent UV effect on cutaneous metalloproteinase activation and cell proliferative response.

## 1. Introduction

Nutlin-3 (NUT) is a cis-imidazoline analog, exhibiting potent antiproliferative activity in cells with functional p53 [1]. Indeed, one of the main functions studied for NUT is its ability to activate p53 through the inhibition of p53–MDM2 complex and prevent cancer development [2,3]. While in cancer cells, NUT has been shown to stimulate apoptotic signaling cascades, in normal cells, such as healthy keratinocytes, the activation of p53 by NUT results in blocking UV-induced apoptosis. For instance, some studies demonstrated that NUT can restore p53 protein expression and activity in aged keratinocyte and can activate p53 in senescent melanoma cells, inducing mitochondrial-mediated apoptosis [4,5]. On the contrary, in normal keratinocytes, NUT may block UV-induced apoptosis by activating p53. Considering that p53 activation has a key role in DNA repair and the subsequent apoptosis in keratinocytes, it is possible that the topical application of NUT could be a good strategy to prevent UV-induced skin damage, including premature skin aging [6]. Despite NUT-potent activity and eventual properties to prevent extrinsic skin damage, its scarce solubility in water and poor bioavailability represents a drawback for its administration, requiring specialized delivery systems able to entrap the molecule in a physiological environment. At this regard, some groups of investigators have encapsulated NUT in nanovesicular systems, such as liposomes or nanoparticulate systems such as polymeric nanoparticles and magnetic solid lipid nanoparticles, for the treatment of hematological malignancies and glioblastoma, as well as breast and prostate cancer [7,8,9,10,11,12,13]. Conversely, to the best of our knowledge, the possibility to treat cutaneous disorders with the topical administration of NUT loaded in nanotechnological delivery systems, has not yet been explored. In this respect, with the aim to find a vehicle suitable for NUT delivery through the stratum corneum barrier, possibly promoting the drug uptake in keratinocytes, in the present investigation lipid vesicular systems typified by ethosomes (ETOs) and transethosomes (T-ETOs) were specifically designed. ETOs are colloidal systems spontaneously forming upon the dispersion of phosphatidylcholine (PC) ethanol solution in water [14]. In ETO dispersions, the PC self-organizes into double-layered spherical vesicles that are able to solubilize especially lipophilic molecules. The high percentages of ethanol (20–45%) distinguish ETOs from liposomes, resulting in longer vesicle stability and higher entrapment of lipophilic drugs, as well as the enhancement of the vesicle penetration potential. Notably, different studies suggested a synergy between PC and ethanol as penetration enhancers, promoting ETO transdermal potential [14,15,16]. Indeed, the PC chemical affinity with the stratum corneum lipids, as well as the ethanol ability to disarrange their organization, endorses the ETO vesicle to pass through the skin. Further advances in vesicle nanotechnology enabled to develop T-ETOs are considered as a new generation of ETOs. T-ETOs are dispersed nanovesicular systems with the same composition of ETO, based on PC, ethanol, and water, enriched with surfactants and used as “edge activators” [17,18,19]. Namely, in T-ETOs the surfactants should change the packing architecture of the PC bilayer, making the vesicles even more elastic and flexible than the ETO ones [19]. A recent transmission electron microscopy study demonstrated that ETOs and T-ETOs can pass through explanted human skin, maintaining their structural integrity, reaching keratinocytes, and thus allowing the intracellular delivery of the loaded drugs [16]. The considerable transdermal potential of ETOs and T-ETOs makes them efficacious drug delivery systems in the treatment of cutaneous pathologies [20,21]. Therefore, besides the formulative approach aimed to produce and characterize NUT-loaded ETO and T-ETO, the present investigation intends also to evaluate their suitability to promote NUT delivery inside the skin. We tested the protective effect of NUT against the cutaneous damage induced by UV radiation. Indeed, due to its location, the skin is the primary barrier of the body against external environments and thus is subjected to the noxious effect of external stimuli, including air pollutants and UV radiations [22,23]. These agents can trigger inflammatory and oxidative stress reactions within the skin (OxInflammation) that promote the onset of different cutaneous disorders, including skin cancer [24,25]. Some cutaneous non-enzymatic and enzymatic molecules can protect the skin, acting as potent antioxidants or oxidant-degrading systems. Regrettably, in some cases, under external stressors, the skin defense mechanisms may not be able to counteract the toxicant destructive effects, resulting in an increase of ROS in the skin, that can induce dermatological diseases, as well as skin aging [26]. In the present study we demonstrate that NUT delivered by ETOs and T-ETOs could prevent the UV-induced damage in human skin, suggesting that these nanotechnological delivery systems may represent a good strategy to counteract the UV-induced skin damage by topical administration of NUT.

## 2. Materials and Methods

### 2.1. Materials

Nutlin-3-a ((-)-4-(4,5-bis(4-chlorophenyl)-2-(2-isopropoxy-4-methoxyphenyl)-4,5-dihydro-1H-imidazole-1-carbonyl) piperazin-2-one, NUT, 98% ee) was synthesized following the procedure reported in the literature [27,28]. Polysorbate 80 (polyoxyethylenesorbitan monooleate, tween 80, T80) was purchased from Sigma-Aldrich (St Louis, MO, USA). The soybean lecithin (PC) (90% phosphatidylcholine) was Epikuron 200 from Lucas Meyer (Hamburg, Germany). STRAT-M^®^ membranes (STRAT-M) were purchased from Merck-Sigma Aldrich (Milan, Italy). Solvents were of HPLC grade, and all other chemicals were of analytical grade.

### 2.2. Ethosome Preparation

A cold method was assessed to prepared the ETOs, as previously described [16]. Briefly, PC at a final concentration of 30 mg/mL was solubilized in ethanol under magnetic stirring at 750 rpm (IKA RCT basic, IKA^®^-Werke GmbH & Co. KG, Staufen, Germany). Then, water was slowly added to the PC-ethanol solution up to 70:30 (*v*/*v*) water/ethanol ratio, under 30 min of stirring. For NUT-loaded ETO preparation, NUT was solubilized in the PC-ethanol solution before adding water. 

### 2.3. Transethosome Preparation 

To prepare the T-ETO, the surfactant T80 was previously solubilized in PC-ethanol solutions (30 or 90 mg/mL), up to a final 1% *w*/*v* concentration. Then, T-ETO preparation was performed by the same method employed for the ETOs, leading to a final T80 0.3% *w*/*w* concentration. For NUT-loaded T-ETO preparation, the drug was solubilized in the PC/T80 ethanol solution before adding water.

### 2.4. Cryo-Transmission Electron Microscopy (Cryo-TEM)

For cryo-TEM investigation, samples of ETO and T-ETO were vitrified by a previously reported method [16]. A Zeiss/Leo EM922 Omega EFTEM Microscopy (Zeiss Microscopy GmbH, Jena, Germany) was employed to examine the specimens with reduced doses ≈1000–2000 e/nm^2^ at 200 kV, by keeping the samples at a temperature below 100 k and a CCD digital camera (Ultrascan 1000, Gatan, Munich, Germany) was used to record the Zero-loss filtered images (ΔE = 0 eV) and the GMS 1.9 software (Gatan, Munich, Germany) was employed to analyze the images. 

### 2.5. Photon Correlation Spectroscopy (PCS) and Zeta Potential Evaluation

A Zetasizer Nano-S90 (Malvern Instr., Malvern, England) was employed to measure the size distribution of the ETOs and T-ETOs, equipped with a 5 mW helium neon laser and a wavelength output of 633 nm. The analyses were conducted at 25 °C at a 90 ° angle and 180 sec run time. Briefly, after diluting the sample with bi-distilled water in a 1:20 *v*/*v* ratio, the size distribution was obtained with the “CONTIN” method [29]. Z average mean diameters ± standard deviation (s.d.) were evaluated. Zeta potential values were measured evaluating the electrophoretic mobility following the Hemholtz–Smoluchowski equation [30].

### 2.6. NUT Entrapment Capacity Evaluation

To evaluate the amount of NUT associated to the ETO and T-ETO, the dispersions were ultrafiltered the day after preparation, as previously reported [19]. The diluted retentate was stirred for 30 min, filtered by a nylon syringe with a 0.22 μm pore diameter and then subjected to HPLC. The filtrate fraction was analyzed by HPLC as such. The entrapment capacity (EC) was determined as follows:

EC = NUT/T_NUT_ × 100 (1)

where NUT stands for the amount of drug retained by the ETO or T-ETO, while T_NUT_ is the total content of NUT employed for the vesicle preparation. At last, the NUT content was quantified by HPLC, as reported below. 

### 2.7. Ethosome and Transethosome Stability Evaluation

The effect of storage on ethosome and transethosome size distribution, as well as on NUT entrapment capacity was investigated on samples kept at 22 °C for 3 months. PCS analyses were performed, as reported above, for 3 months after ETO and T-ETO preparation, EC ± s.d. was also evaluated on the same samples.

### 2.8. In Vitro Permeation Test (IVPT)

Diffusion vertical cells (0.9 cm orifice diameter; PermeGear Inc., Hellertown, PA, USA) were employed joined to STRAT-M [31]. Five milliliters of ethanol/water 50:50, *v*/*v* were added to the cell receptor compartment to obtain sink conditions. During the entire experiment, a magnetic stirring of 500 rpm was kept and a constant temperature of 32 ± 1 °C was maintained [32]. The donor compartment was filled with 500 microliters of NUT-loaded ETO, NUT-loaded T-ETO, or NUT ethanolic solution (ethanol/water 30:70, *v*/*v*) at a concentration of NUT 0.3 mg/mL (SOL-NUT) and the donor compartment was sealed to avoid evaporation of the samples. Five hundred microliters of receptor phase were collected at predetermined time intervals (1–24 h) and analyzed by HPLC. At each time interval, the receiving compartment was refilled with an equal volume of receptor phase. The concentration of NUT was analyzed 6 times for every independent experiment conducted and expressed as the mean values ± s.d. The amount of NUT retained by the membranes were evaluated at the end of the experiments by cutting the membranes into small pieces that were then placed in vials containing 1 mL of ethanol (Branson, Bransonic^®^ M Mechanical Bath 3800, Emerson, St. Louis, MO, USA) and ultrasonicated for 15 min. Nylon syringe membranes were used to filter the obtained suspension that was therefore analyzed by HPLC to evaluate the NUT content (M_NUT_). 

For data evaluation, the amount of NUT permeated (μg/cm^2^) was plotted as a function of time. The IVPT parameters were calculated as previously reported [19], according to the Fick’s law that describes the steady-state permeation through the skin, considering negligible, under sink conditions, the drug concentration in the receptor compartment, with respect to that in the donor compartment [33,34]. 

### 2.9. HPLC Analysis

The Perkin Elmer, Series 200 HPLC Systems supplied with a micro-pump, an auto sampler, and a UV-detector operating at 255 nm was employed to conduct the HPLC analysis. A stainless-steel C-18 reverse-phase column (15 × 0.46 cm) packed with 5 μm particles (Hypersil BDS C18 Thermo Fisher Scientific S.p.A., Milan, Italy) was used. The mobile phase adopted was acetonitrile/water 40:60 *v*/*v*, pH 3, eluted at 1 mL/min flow rate. The sample injection volume was 5 μL, while the retention time was 9 min.

### 2.10. Ex Vivo Studies

#### 2.10.1. Skin Explants Culturing and Treatment

Skin explants were obtained from elective abdominoplasties purchased from HKB Surgery Hospital in Huntersville, NC, as previously described [24]. Briefly, upon arrival, the subcutaneous fat was trimmed from the skin and 12 mm punch biopsies were collected and cultured [24]. After the overnight recovery in the incubator at 37 °C, 5% CO_2_, skin explants were pretreated with the different SOL, ETO, and T-ETO formulations loaded with NUT (NUT_0.3_) at the dose of 10 µM, applying 20 uL of the dispersions diluted in PBS. The vehicles were spread onto the skin explants with a glass rod. Skin samples were incubated for 24 h at 37 °C and then pretreated each day for 3 days with different dispersions. After each treatment, skin biopsies were exposed twice a day to 200 mJ UVA/UVB using a solar simulator, as previously described [35]. Samples were exposed to UV each day for 3 days and collected after the last day of exposure (DAY4) for the subsequent analysis. Three different donors were employed to conduct the experiments. 

#### 2.10.2. LDH Assay

Lactate Dehydrogenase (LDH) toxicity assay was conducted on the media of the skin explants to test their viability upon the treatment with the different vehicles. Media of the skin explants was collected 24 h and 4 days after the first treatment with SOL, ETO, and T-ETO formulations loaded with NUT_0.3_. According to the manufacturer’s instructions (Roche, Indianapolis, IN, USA, cat no. 11644793001), the amount of lactate dehydrogenase (LDH) enzyme released in the media of the skin samples was measured to evaluate the cytotoxicity of the different formulations, as previously described [36]. Levels of LDH released into the media were normalized to the negative control considered as the 100% of skin explants viability.

#### 2.10.3. Immunohistochemistry

Immunohistochemistry was performed on 4 mm of skin biopsies sections, as previously described [37]. Sections were incubated with primary antibodies ki67 (cat HCA053, Bio-Rad Laboratories, Inc., Hercules, CA, USA) at 1:200 (*v*/*v*) dilution and MMP9 (NBP2-13173, Novus Biological, Littleton, CO, USA) at 1:100 (*v*/*v*) dilution in 0.25% BSA/PBS overnight at 4 °C. The next day, sections were incubated with the fluorochrome-conjugated secondary antibody (A11004 Alexa Fluor 568, Invitrogen, ThermoFisher Scientific, Waltham, MA, USA) at 1:1000 (*v*/*v*) dilution in 0.25% BSA/PBS for 1 h at 22 °C. The nuclei were stained with DAPI (D1306, Invitrogen, ThermoFisher Scientific, Waltham, MA, USA) for 1 min in PBS at RT. FluoroG mounting media (ThermoFisher Scientific, Waltham, MA, USA) was used to mount the sections onto glass slides and a Zeiss LSM10 microscope was employed to acquire images of the skin sections at 40× magnification. Images were quantified using ImageJ 1.5.3 software.

#### 2.10.4. Statistical Analysis

The statistical analyses were conducted by GraphPad Prism 6 (GraphPad Software Inc., La Jolla, CA, USA) adopting analysis of variance (1-way or 2-way ANOVA), followed by Tukey’s post hoc test. Statistical significance was taken at *p* < 0.05 for all variables tested. Data are expressed as mean ± SD of three independent experiments.

## 3. Results

### 3.1. Preparation of Ethosomes and Transethosomes

Lipid vesicular systems constituted of PC were designed as topical vehicles for NUT solubilization and application on the skin. Particularly, since NUT is insoluble in water and slightly soluble in ethanol (5.69 mg/mL), ETOs and T-ETOs were chosen, being obtained upon addition of water to ethanolic solutions of PC under stirring. Particularly, PC 0.9%, and ethanol 30% were selected based on previous studies [38,39]. For NUT loading in ETO, different drug concentrations were considered, namely 0.01, 0.015 and 0.03%, *w*/*w* (Table 1). In all cases, where milky dispersions free from separation phenomena were obtained. The presence of NUT did not affect the ETOs aspect. In the case of T-ETO, the composition was improved by adding to the PC ethanolic solution the non-ionic surfactant T80 (0.3% *w*/*w*), resulting in more translucent dispersions. The T80 concentration was previously found suitable to produce stable and homogeneously sized vesicles [16,40,41].

### 3.2. Size, Zeta Potential, and Morphology 

To gain information about the size distribution of the ETO and T-ETO, PCS was employed. As summarized in Table 2, vesicle mean diameters, expressed as Z Average, ranged between 140 and 240 nm, 1 day after preparation. Some typical size distribution plots of ETO and T-ETO are reported in Figure S1.

In the case of ETOs, the NUT presence slightly affected mean diameters. For this reason, the highest NUT concentration (0.03%) was selected also for T-ETO loading. In general, the presence of T80 reduced the mean diameters. Namely, in the case of empty vesicles, Z Average of T-ETO was almost 70 nm lower with respect to ETOs. The mean diameter of T-ETO-NUT_0.3_ was slightly smaller with respect to empty T-ETO (Table 2). Remarkably, the polydispersity indexes below 0.2 indicate homogeneous size distributions, mostly characterized by the presence of one peak, as shown in Figure S1. 

Electrophoretic light scattering enabled to measure the surface charge of vesicles, expressed as zeta potential. The ETO and T-ETO displayed negative zeta potential values (Table S1), resulting in an electrostatic repulsion, due to ethanol that confers negative charges to the PC polar head groups [42]. In the presence of NUT, zeta potential values became more negative, with an increase of absolute value around nine, both in the case of ETO-NUT and T-ETO-NUT, suggesting that the drug further enables to avoid vesicle aggregation. 

**Figure 1:**
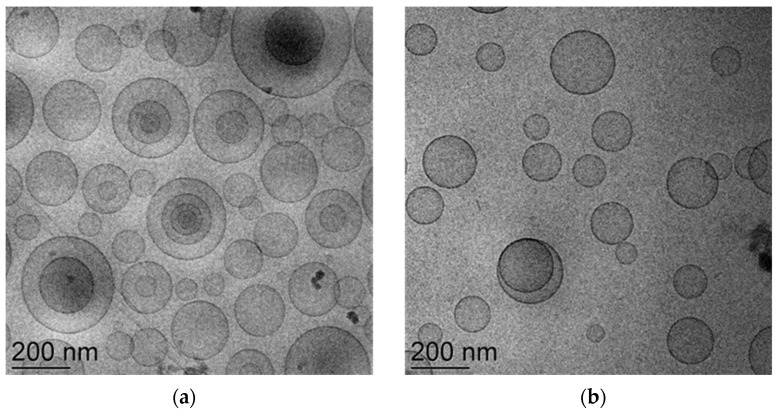
Cryo-TEM images of ETO-NUT_0.3_ (**a**), and T-ETO-NUT_0.3_ (**b**). The bars equal 200 nm.

Regarding morphology, Figure 1 reports cryo-TEM images of ETO-NUT_0.3_ and T-ETO-NUT_0.3._

As shown in the micrographs, the PC double layer organization gave rise to spherical vesicles, particularly the ETO-NUT_0.3_ vesicles were oligolamellar (Figure 1a), while in the case of T-ETO-NUT_0.3_ unilamellar vesicles were detectable (Figure 1b), suggesting that the surfactant T80 slightly affected the vesicle structures.

### 3.3. NUT Entrapment Capacity (EC)

To evaluate the EC of NUT in the ETO and T-ETO, the formulations were subjected to ultrafiltration to separate the lipid phase from the aqueous phase. Then, the vesicle disaggregation was promoted by dissolving them in ethanol. Table 3 reports the EC results. The HPLC quantification of NUT in both phases confirmed the almost total recovery of the drug in the disperse phase, with similar results for ETO-NUT_0.3_ and T-ETO-NUT_0.3_ (EC day 1). Figure S2 shows the HPLC standard calibration curve for NUT.

### 3.4. Stability Studies

**Figure 2:**
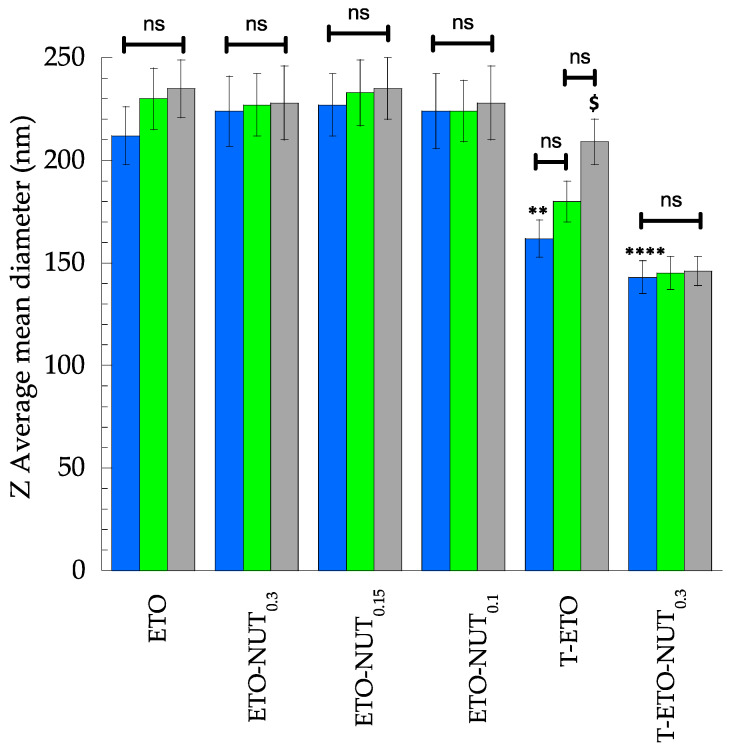
The effect of storage on size of ethosomes (ETOs) and transethosomes (T-ETOs) kept at 22 °C for 3 months. Z Average mean diameters (*n* = 3) were measured by PCS 1 (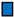) 60 (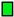) and 90 (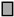) days after preparation. ** *p* < 0.01 T-ETO D1 vs. ETO D1; **** *p* < 0.001 T-ETO-NUT_0.3_ D1 vs. ETO D1 and $ *p* < 0.05 T.ETO D90 vs. T-ETO D1 by 2-way ANOVA followed by Tukey’s post hoc comparison test; ns: not significant.

The effect of storage on vesicle size and EC of NUT was investigated on nanovesicular forms kept at 22 °C for 3 months (Table 2, Figure 2). 

Vesicle size was quite stable, especially in the case of NUT-loaded vesicles. Indeed, NUT appeared to stabilize the vesicle mean diameter. Notably, the T-ETOs underwent a 47 nm increase within 90 days, passing from 162 to 209 nm (Table 2), while in the presence of NUT_0.3_, vesicle mean diameter was smaller and kept almost unvaried by time (Table 2). The EC value of NUT in ETO-NUT_0.3_ and T-ETO-NUT_0.3_ after 3 months (EC _day 90_) was around 72% for both kind of nanovesicle systems. Thus, the EC decrease was 17% and 19%, for ETO-NUT_0.3_ and T-ETO-NUT_0.3_, respectively.

### 3.5. IVPT 

To investigate the influence of nanovesicular systems on NUT permeation, diffusion cells were associated to the synthetic STRAT-M. This multi-layered polymeric membrane system has been demonstrated as suitable to mimic the stratum corneum. It is indeed made of two polyether sulfone layers overlapped to one polyolefin bottom layer, resulting in a skin-like, tortuous, porous structure [31]. The impregnation with synthetic lipids further yields this membrane system a stratum corneum affinity, conferring barrier properties due to the coexistence of both hydrophilic and lipophilic compartments.

Two main steps can be distinguished to model the passive kinetic process of a drug permeating from the vehicle into the skin or into membrane layers [34]. The first step is related to the behavior of the drug releasing from its vehicle and to its preferential distribution in the skin/membrane or in the vehicle. This behavior is described by the partition coefficient (P). The second step refers to the further diffusion of the drug through the lipophilic/hydrophilic layers of the skin/membrane, depending on the chemical properties of the drug and on the vehicle composition. The diffusion coefficient (D) and the permeability coefficient (Kp) are the parameters characterizing this latest step. 

**Figure 3:**
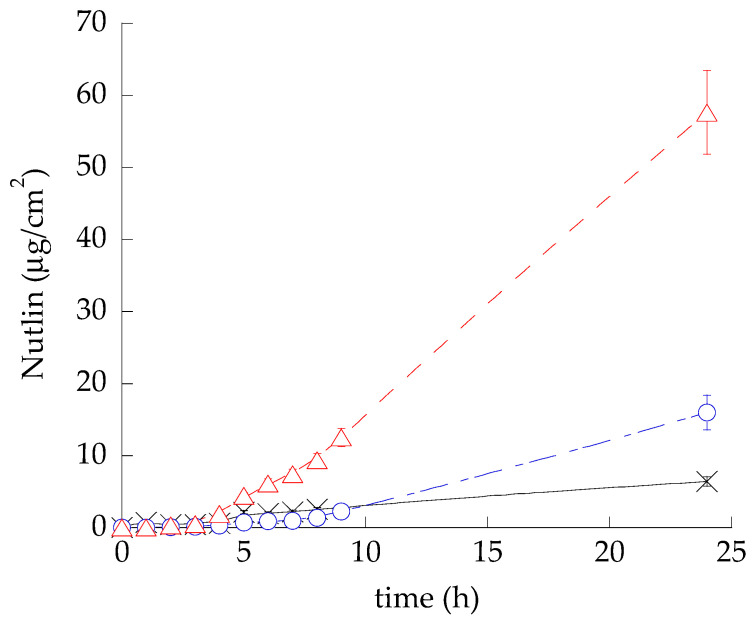
NUT permeability kinetics from ETO-NUT_0.3_ (blue circles, chain line), T-ETO-NUT_0.3_ (red triangles, dotted line), and SOL-NUT_0.3_ (black crosses, continuous line), as determined by Franz cell associated to STRAT-M. Data are the mean of six independent experiments ± s.d.

Figure 3 shows the permeation profiles of NUT through STRAT-M for all formulations, while Table 4 reports the fluxes (Jss) and other permeation parameters.

**Figure 4:**
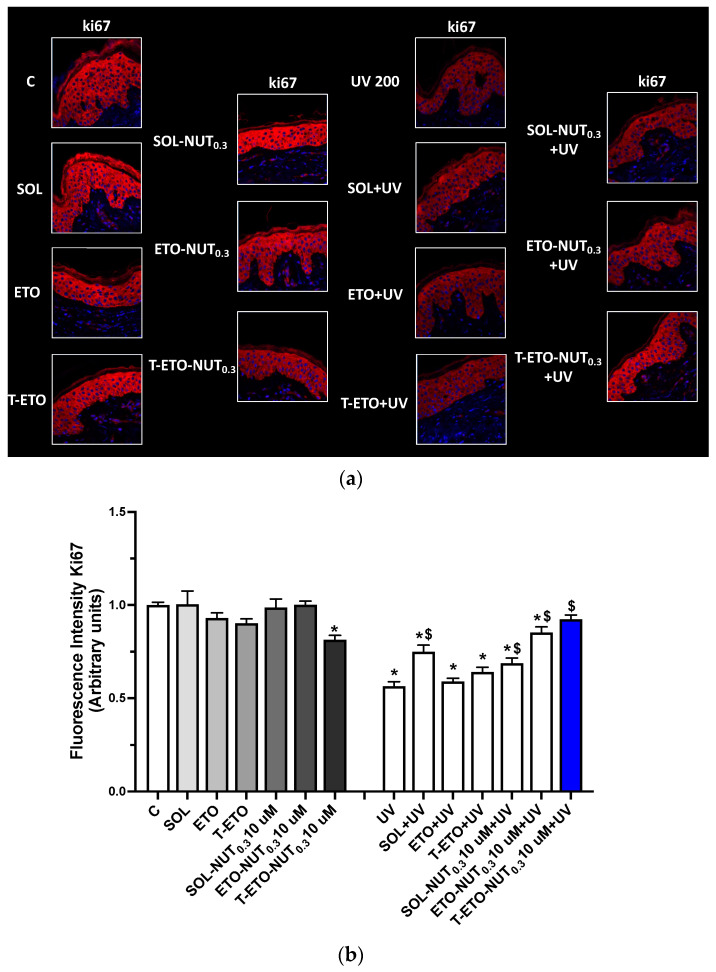
(**a**) Ki67 expression levels (red staining) in human skin biopsies treated with unloaded forms (SOL, ETO, T-ETO) or loaded with NUT_0.3_ (SOL-NUT_0.3,_ ETO-NUT_0.3,_ T-ETO-NUT_0.3_) at the dose of 10 µM for 4 days and exposed to UV 200 mJ/cm^2^ for 3 days. Blue staining (DAPI) represents nuclei. (**b**) Graphical representation of the fluorescence intensity levels of ki67 quantified using ImageJ software; original magnification at 40×. The data are expressed as the mean of three different experiments; * *p* < 0.05 T-ETO-NUT_0.3_ 10 µM, UV, SOL-UV, ETO + UV, T-ETO + UV, SOL-NUT_0.3_ 10 µM + UV, ETO-NUT_0.3_ 10µM + UV) vs. C; ^$^ *p* < 0.05 SOL + UV, ETO-NUT_0.3_ 10 µM + UV, T-ETO-NUT_0.3_ 10 µM + UV vs. UV by 2-way ANOVA followed by Tukey’s post hoc comparison test.

The fastest NUT kinetic was detected in the case of T-ETO-NUT_0.3_, followed by SOL-NUT_0.3_ and ETO-NUT_0.3_ (Figure 4), as reflected by the Kp of T-ETO-NUT_0.3_, that was 7.4-fold higher, with respect to ETO-NUT_0.3._ Remarkably, the D values of NUT loaded in ETO and T-ETO were, respectively, 240- and 260-fold higher, with respect to the drug in solution. In addition, the *p* values of NUT were dramatically lower in the case of vesicular systems with respect to the drug solution, suggesting that NUT was strongly associated within PC vesicles. Moreover, the P coefficient of ETO-NUT_0.3_ was 9-fold lower with respect to T-ETO-NUT_0.3_, indicating that the composition of vesicular system affects NUT affinity for the vesicles. Particularly the presence of T80 promotes the partition of NUT towards the membrane. The amount of NUT diffused after 24 h (A_NUT_ total) confirmed the capability of T-ETO-NUT_0.3_ to improve NUT permeability, with respect to ETO-NUT_0.3_ or to the simple drug solution. Indeed, in the case of T-ETO-NUT_0.3_, the improvement ratio of A_NUT_ total was 3.6 and 9 with respect to ETO-NUT_0.3_ and SOL-NUT_0.3_, respectively. Statistical differences between Kp values were extremely significant (*p* < 0.0001).

### 3.6. Evaluation of the Protective Effects against UV-Induced Skin Damage in Human Skin 

#### 3.6.1. Immunofluorescence Staining Analysis of Cell Proliferation (ki67)

The protective role of ETOs and T-ETOs loaded with NUT_0.3,_ was tested against the skin damage induced by UV radiations. Human skin is everyday subjected to the harmful effects of different environmental insults, among which UV radiations represent the most dangerous stimuli. In this respect, to mimic the natural UV exposure, human skin biopsies were treated with ETO-NUT_0.3_ and T-ETO-NUT_0.3_ and subjected to UV radiations. First, an LDH assay was assessed on skin explants treated for 24 h (DAY1) for up to 4 days (DAY4) with SOL, ETO, and T-ETO containing or not, NUT_0.3_ at the dose of 10 µM to evaluate the possible cytotoxic effect of the different nanovesicular systems on skin viability (Figure S3). The treatment dose of 10 µM was selected based on preliminary results obtained on primary keratinocytes. Either the NUT_0.3_-loaded or -unloaded nano-formulations displayed any significant effect on the skin explants viability (Figure S3). Therefore, to evaluate the protective effect of NUT_0.3_ nanovesicular systems against skin damage, the level of ki67 was measured as marker of cell proliferation in skin tissues exposed to UV and pretreated with SOL NUT_0.3_, ETO-NUT_0.3_ and T-ETO-NUT_0.3_ at the selected dose of 10 µM. As depicted in Figure 4a,b UV radiation induced a decrease in Ki67 levels in human skin biopsies, indicating a reduced cells proliferation rate compared to control tissues. Unloaded SOL, ETO, and T-ETO did not significantly affect Ki67 expression levels, whereas the same formulations containing NUT_0.3_-loaded nanovesicular systems could prevent ki67 downregulation induced by UV exposure. Of note, skin explants treated with T-ETO-NUT_0.3_ resulted in completely restored levels of ki67 compared to unexposed tissues, suggesting the potential of T-ETO to enhance NUT delivery into the skin.

#### 3.6.2. Immunofluorescence Staining Analysis of Metalloproteinase MMP9 Activation

**Figure 5:**
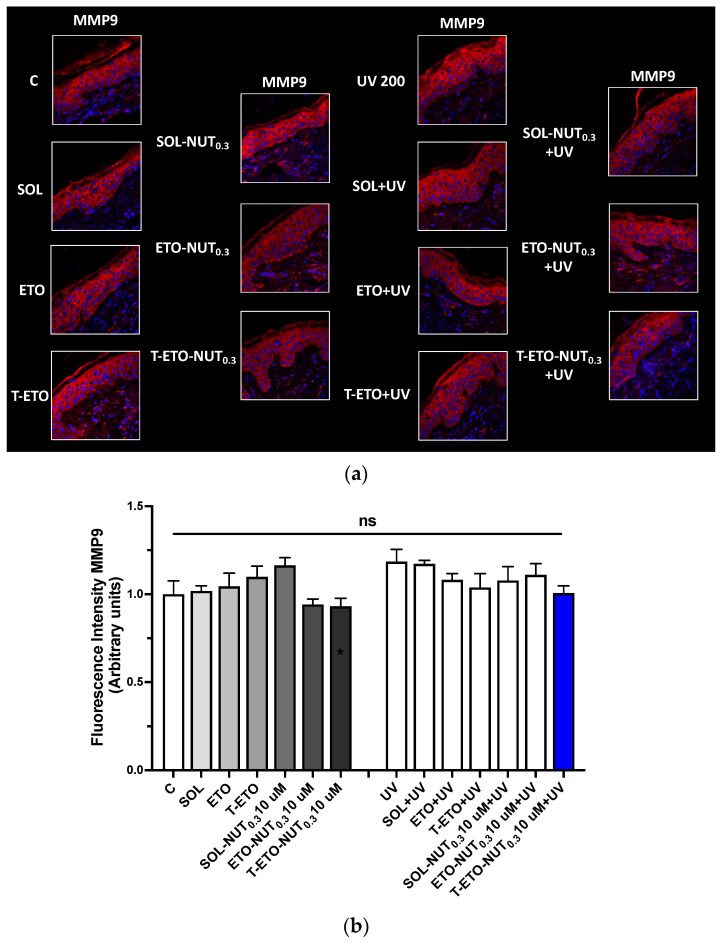
(**a**) Representative pictures of MMP9 fluorescence in skin explants treated with unloaded forms (SOL, ETO, T-ETO) or loaded with NUT_0.3_ (SOL-NUT_0.3,_ ETO-NUT_0.3,_ T-ETO-NUT_0.3_) at the dose of 10 µM for 4 days and exposed to 200 mJ/cm^2^ of UV for 3 days. Red staining represents MMP9, Blue staining (DAPI) represents nuclei. (**b**) Graphical representation of MMP9 fluorescence intensity levels quantified using ImageJ software; original magnification at 40×. The data are expressed as the mean of three different experiments; no significance (ns) between all the conditions was detected by 2-way ANOVA followed by Tukey’s post hoc comparison test.

UV radiation can promote the activation of metalloproteinases (MMPs) as MMP9, a proteolytic enzyme implicated in the degradation of proteins belonging to the extracellular matrix (ECM) as collagen, elastin, etc., resulting in skin structure damage and premature extrinsic aging [43,44]. Therefore, we tested whether the NUT-loaded nanovesicular systems could prevent UV-induced MMP9. Our results show that exposure to UV radiation could promote a slight activation of MMP9 compared to control tissues, as depicted by increased fluorescent levels of the proteolytic enzyme (Figure 5a,b). Notably T-ETO-NUT_0.3_ could restore MMP9 levels at basal condition, whereas SOL-NUT_0.3_ and ETO-NUT_0.3_ did not display any protective effect against the UV damage. This data might indicate that T-ETO compared to ETO could represent a promising delivery system to deliver NUT_0.3_ within the skin to protect UV-induced skin aging. 

## 4. Discussion

Skin aging is an inevitable biological phenomenon of human life. Advancing age brings changes to our cutaneous tissues. Since the skin is an interface between the body and the environment, it is continuously exposed to environmental stressors, representing the main cause of cutaneous extrinsic aging (photo-aging). Indeed, since it has been thoroughly demonstrated that photo-aging is a consequence of exposure to ultraviolet radiations [45,46], in the last few decades, the skin care market based on natural or synthetized products is constantly growing. Particularly, in recent years, drugs originally developed for different therapeutic indications have been demonstrated to be suitable in the treatment of aging, such as rapamycin and metformin [47,48]. In addition, new delivery systems are continuously under investigation to improve skin uptake of bioactive compounds. Indeed, the delivery of new molecules inside the skin strata can be challenging because of the peculiar anatomical skin structure that represents a barrier, with respect to drug delivery.

In this respect, ETOs and T-ETOs were specifically designed for NUT loading, being lipid based nanovesicular systems characterized by high biocompatibility and transdermal potential. These disperse systems are formed by the spontaneous double-layer organization of PC in water, resulting in vesicles suitable to solubilize lipophilic molecules, such as NUT. Furthermore, ethanol can induce disorder on cell membranes, thus promoting the passage of ETOs and T-ETOs, as previously demonstrated [20,21]. The simple ETO and T-ETO preparation approach described here avoids the use of toxic organic solvents and high temperature, is eco-sustainable and cost effective since it requires low energy, and lastly it can be easily scaled-up. 

The presence of ethanol enabled us to efficiently solubilize the NUT and to strongly maintain the drug associated to the PC vesicles. The vesicle composition could account for their different architecture; oligolamellar in the case of ETO-NUT_0.3_, unilamellar in the case of T-ETO-NUT_0.3_ (Figure 1). In this latest case, it is likely that T80 oleate chains intercalate within the lipid bilayer, resulting in an alteration of the lamellar packing organization, as well as in a reduction of the vesicle mean diameter. The presence of NUT further decreased and stabilized T-ETO-NUT_0.3_ mean diameter (Table 2) and their ionic environment, as indicated by the more negative zeta potential value (Table S1), probably because of its positioning at the interface between the vesicles and the dispersing phase, as previously found in a study about dimethyl fumarate-loaded T-ETO [19]. 

IVPT results conducted using STRAT-M, mimicking the stratum corneum structure, suggested that both ETO-NUT_0.3_ and T-ETO-NUT_0.3_ were able to enhance the drug diffusion through the membrane, with respect to SOL-NUT (Figure 3). The ethanol solution of NUT provided the highest drug concentration in the membrane and the highest *p* value, reflecting the affinity of the drug in solution towards the STRAT-M membrane that mimics the *stratum corneum*. Two important points should be mentioned: (i) in the case of vesicular systems, the diffusion and permeation of NUT is instead increased with respect to the plain solution, suggesting the capability of the nanovesicular systems to promote the passage of the drug through the *stratum corneum*; (ii) ethanol solution is not a physiological vehicle, when applied onto the skin it could induce irritation and skin dryness, thus it cannot be topically applied. Notably T-ETO-NUT_0.3_ greatly improved A_NUT_ with respect to SOL-NUT and ETO-NUT_0.3_ (Table 3), confirming the capability of the unilamellar vesicles formed by the T80 association with PC to enhance NUT permeability. It is noteworthy that T-ETO can pass intact through the *stratum corneum*, maintaining their structure in the upper strata, such as *stratum corneum* and *granulosum*, while their presence decreases in the deeper skin layers [16]. Thus, a very low vesicle percentage can reach the systemic circulation, resulting in NUT delivery confined into the skin strata.

Notably, the ability of ETOs and T-ETOs to deliver NUT through the human skin and the possible protective role of this molecule against the UV-induced skin damage was evaluated. Thus, human skin biopsies were exposed to UV radiations up to 4 days, two times per day, to simulate a daily sun exposure. In this context, two different markers of UV-induced skin damage as Ki67, a cells proliferation marker, and MMP9, a metalloproteinase involved in extracellular matrix (ECM) degradation, were evaluated by assessing immunofluorescence staining analysis on human skin tissues. We found that, when loaded in ETO, and more evidently in T-ETO, NUT could significantly protect the loss in cells proliferation induced by UV exposure, restoring the cutaneous basal levels of the cell proliferation marker ki67 (Figure 4). 

Interestingly, some studies demonstrated that NUT could prevent UVB-induced skin photodamage by promoting DNA damage repair via p53 activation and thus diminishing apoptosis. For instance, human keratinocytes treated with NUT displayed increased levels of p53 associated with enhanced long-term cell survival, as well as attenuated DNA damage response (DDR) upon UV radiations [49], while topical application of NUT was found to decrease thymine dimers formation, a marker of DNA damage, and apoptosis in keratinocytes of hairless mice [50].

In addition, we also found that T-ETO-NUT_0.3_, and in a less extensive way, ETO-NUT_0.3_, could prevent the slight increase in metalloproteinase MMP9 expression, involved in ECM degradation (Figure 5). Indeed, along with DNA damage and carcinoma, a prolonged exposure to UV radiations has been shown to promote a pro-inflammatory environment called “senescence-associated secretory phenotype (SASP)”, relying on the secretion of many pro-inflammatory mediators that can favor extracellular matrix (ECM) degradation, as well as cell senescence and apoptosis [51]. In particular, the activation of proteolytic enzymes as metalloproteinases (MMPs) in response to UV radiations can lead to the degradation of extracellular matrix and dermal proteins (elastin, collagen, etc.); thus, favoring premature skin aging and the establishment of a microenvironment suitable for tumor progression and metastasis [43,44]. 

Considering that ECM degradation is a long process occurring in response to a prolonged sun exposure, the increase in MMP9 expression found in UV exposed skin explants, although not significant compared to control, may suggest the initial activation of the skin aging process. Indeed, we suggest that a prolonged exposure of the skin samples to UV radiation might promote a significant induction of MMP9, leading to ECM degradation and related skin damage in the long term. In addition, we found that the formulations containing NUT could restore MMP9 basal levels, suggesting that NUT may exert a protective role against skin damage. Also, in this context T-ETO-NUT_0.3_ demonstrated to be more suitable to deliver NUT through the skin tissue, with respect to ETO-NUT_0.3_ and SOL-NUT_0.3_, confirming the IVPT findings. 

## 5. Conclusions

Taken together, our results showed that NUT could prevent the UV-induced damage by protecting from the possible cell death and ECM degradation; thus, suggesting a protective role against premature skin aging and the development of a microenvironment suitable for cancer progression in the long term. Since NUT has been demonstrated to target many types of hematological malignancies, as well as solid tumors (i.e., breast cancer, retinoblastoma, prostate cancer, lymphoma, and melanoma), this preliminary study suggests that T-ETO may represent a promising delivery system for NUT, possibly able to protect the skin from UV damage. Indeed, this approach aims to protect the skin “from the inside out”, as once the drug is permeated, it could be able to prevent the UV damage at each skin layer. Nevertheless, further studies are required to understand the potential applicability of T-ETOs in the treatment of skin aging. Moreover, since T-ETO-NUT_0.3_ topical delivery could imply to some extent NUT systemic distribution, further studies will be conducted to evaluate the drug plasmatic concentration upon skin administration of T-ETO-NUT_0.3_ in mice.

## Figures and Tables

**Table 1:** Composition of the indicated formulations.

Formulation	PC ^1^	Ethanol	T80 ^2^	Water	NUT ^3^
Code	% *w*/*w*	% *w*/*w*	% *w*/*w*	% *w*/*w*	% *w*/*w*
ETO	0.900	29.100	-	70.00	-
ETO-NUT0.3	0.900	29.070	-	70.00	0.030
ETO-NUT0.15	0.900	29.085	-	70.00	0.015
ETO-NUT0.1	0.900	29.090	-	70.00	0.010
T-ETO	0.900	29.100	0.300	69.70	-
T-ETO-NUT0.3	0.900	29.070	0.300	69.70	0.030

^1^: soy phosphatidylcholine; ^2^: polysorbate 80; ^3^: Nutlin-3a.

**Table 2:** Size distribution parameters of ethosomes and transethosomes, as determined by PCS.

Formulation Code	Time (Days)	Z Average (nm) ± s.d.	Typical Intensity Distribution		Polydispersity Index ± s.d.
			nm *	Area (%) *	
ETO	1	212.25 ± 14.2	237.6	98.9	0.12 ± 0.01
	90	235.22 ± 20.5	245.5	100.0	0.14 ± 0.03
ETO-NUT_0.3_	1	224.2 ± 11.57	245.1	99.0	0.17 ± 0.04
	90	228.9 ± 19.42	242.4	99.1	0.14 ± 0.03
ETO-NUT_0.15_	1	227.2 ± 10.78	233.5	95.8	0.20 ± 0.02
	90	234.6 ± 15.55	250.7	97.6	0.17 ± 0.04
ETO-NUT_0.1_	1	224.4 ± 10.60	232.9	96.9	0.20 ± 0.01
	90	228.3 ± 15.30	236.2	97.4	0.16 ± 0.04
T-ETO	1	161.90 ± 11.32	148.7	99.6	0.13 ± 0.02
	90	209.10 ± 18.22	198.7	100.0	0.16 ± 0.03
T-ETO-NUT_0.3_	1	143.1 ± 14.88	169.0	99.8	0.15 ± 0.04
	90	146.5 ± 13.52	162.1	100.0	0.14 ± 0.03

s.d.: standard deviation; *: main peak; data are the mean of three independent determinations on different batches.

**Table 3:** Entrapment capacity of the indicated forms.

Formulation Code	EC ^1^ _day 1_ (%)	EC ^1^ _day 90_ (%)
ETO-NUT_0.3_	89.93 ± 6.70	72.62 ± 8.33
T-ETO-NUT_0.3_	91.64 ± 5.22	72.58 ± 4.21

^1^: Entrapment capacity, as defined in Equation (1), evaluated the day after nanovesicle preparation (day 1) or after 3 months (day 90) of storage; data are the mean of three independent experiments ± s.d.

**Table 4:** IVPT parameters of the indicated forms, determined by Franz cell associated to STRAT-M.

Formulation Code	Jss ^1^ (μg cm^−2^ h^−1^)	T_lag_ ^2^ (h)	Kp ^3^ (cm h^−1^ 10^−3^)	D ^4^ (cm^2^ h^−1^) × 10	P ^5^ STRAT-M/Vehicle	A_NUT_ ^6^ (μg cm^−2^)	M_NUT_ ^7^ (μg cm^−2^)
ETO-NUT_0.3_	0.24 ± 0.12	2.52 ± 0.10	0.81 ± 0.36	16.80 ± 0.10	0.01 ± 0.02	15.9 ± 12.1	7.9 ± 7.5
T-ETO-NUT_0.3_	1.79 ± 0.42	2.75 ± 0.12	5.98 ± 1.24	18.33 ± 0.12	0.09 ± 0.01	57.6 ± 18.2	9.8 ± 6.0
SOL-NUT_0.3_	0.44 ± 0.02	2.00 ± 0.12	1.49 ± 0.06	0.07 ± 0.01	5.96 ± 0.02	6.4 ± 0.5	24.3 ± 6.2

^1^: steady state flux per unit area, ^2^: lag time; ^3^: permeability coefficient; ^4^: diffusion coefficient; ^5^: partition coefficient; ^6^: cumulative amount of NUT diffused at 24 h; ^7^: NUT associated to the membrane after 24 h; data are the mean of six independent Franz cell experiments ± s.d.

## Data Availability

The datasets generated during and/or analyzed during the current study are available from the corresponding author on reasonable request.

## Supplementary Materials

The following supporting information can be downloaded at: https://www.mdpi.com/article/10.3390/life14010155/s1, Figure S1: Typical size distribution plots corresponding to samples of ETO (a), ETO-NUT_0.3_ (b), T-ETO (c), and T-ETO-NUT_0.3_ (d), as obtained by PCS; Table S1: Zeta potential values of the indicated vesicular systems; Figure S2: HPLC calibration curve of standard NUT obtained by the reported conditions; Figure S3: LDH assay on skin biopsies treated with SOL, ETO and T-ETO unloaded or loaded with NUT_0.3_ at the dose of 10 µM for 1 day (DAY 1) up to 4 days (DAY 4).
