# Supplementary material for: Nutlin-3 Loaded Ethosomes and Transethosomes to Prevent UV-Associated Skin Damage
Source: Life (Basel). 2024 Jan 21;14(1):155. doi: 10.3390/life14010155 (PMC10817472; doi:10.3390/life14010155)
Supplement: Supplementary file 1 [file life-14-00155-s001.zip › life-2745828-supplementary.pdf]

life

MDPI

Supplementary Materials

Nutlin-3 Loaded Ethosomes and Transethosomes to Prevent UV-Associated Skin Damage

Elisabetta Esposito [1,*], Francesca Ferrara [1], Markus Drechsler [2], Olga Bortolini [3], Daniele Ragno [1], Sofia Toldo [3], Agnese Bondi [1], Alessandra Pecorelli [3], Rebecca Voltan [3], Paola Secchiero [4], Giorgio Zauli [5] and Giuseppe Valacchi [3,6,*]

1 Department of Chemical, Pharmaceutical and Agricultural Sciences, University of Ferrara, I-44121 Ferrara, Italy; frrfnc3@unife.it (F.F.); daniele.ragno@unife.it (D.R.); agnese.bondi@unife.it (A.B.)
2 Bavarian Polymer Institute (BPI) Keylab "Electron and Optical Microscopy", University of Bayreuth, D-95440 Bayreuth, Germany; mardre@mardre.com
3 Department of Environmental Sciences and Prevention, University of Ferrara, I-44121 Ferrara, Italy; olga.bortolini@unife.it (O.B.); sofia.toldo@unife.it (S.T.); alessandra.pecorelli@unife.it (A.P.); rebecca.voltan@unife.it (R.V.)
4 Department of Translational Medicine and LTTA Centre, University of Ferrara, I-44121 Ferrara, Italy; paola.secchiero@unife.it
5 Research Department, King Khaled Eye Specialist Hospital, Riyadh 11462, Saudi Arabia; giorgio.zauli@unife.it
6 Plants for Human Health Institute, Animal Sciences Department, NC Research Campus, NC State University, Kannapolis, NC 28081, USA
* Correspondence: ese@unife.it (E.E.); vlcgpp@unife.it (G.V.)

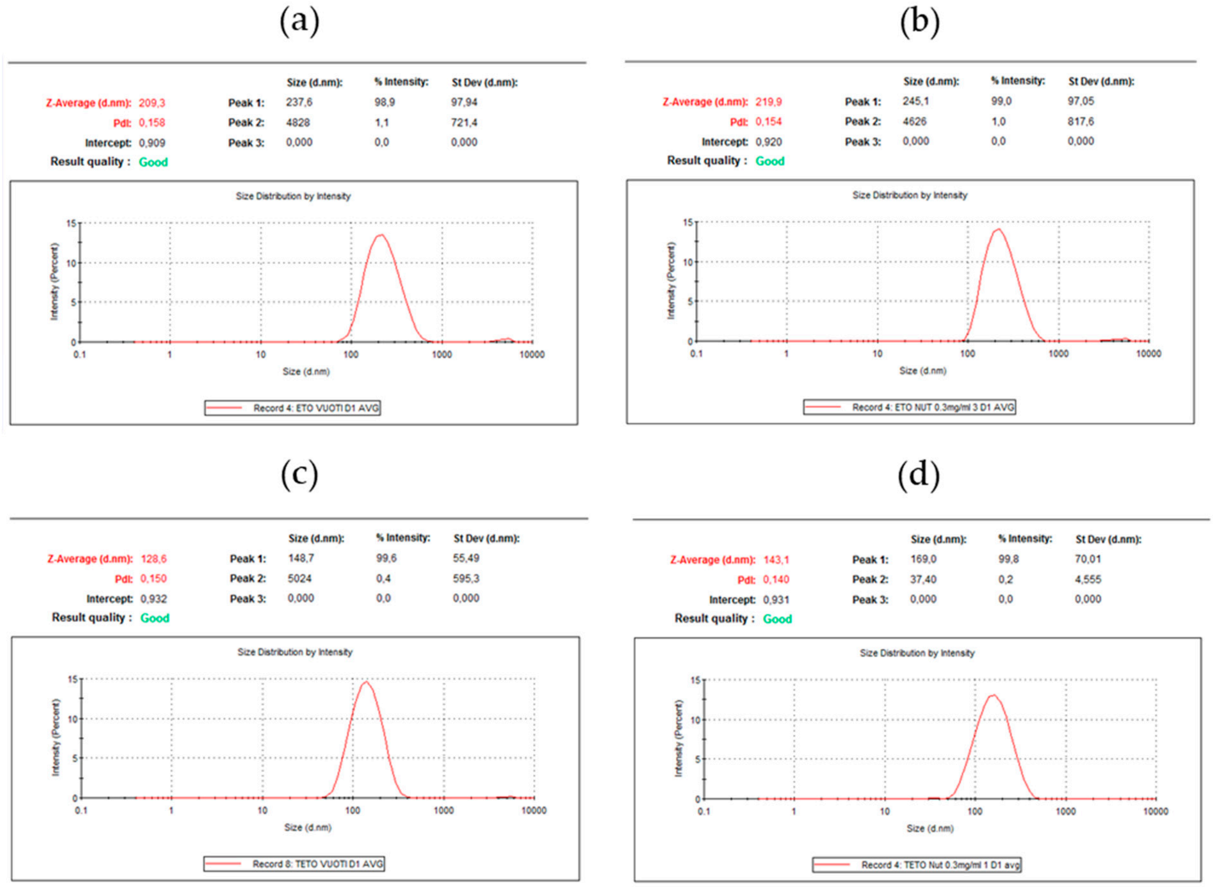

Figure S1. Typical size distribution plots corresponding to samples of ETO (**a**), ETO-NUT$_{0.3}$ (**b**), T-ETO (**c**), and T-ETO-NUT$_{0.3}$ (**d**), as obtained by PCS.

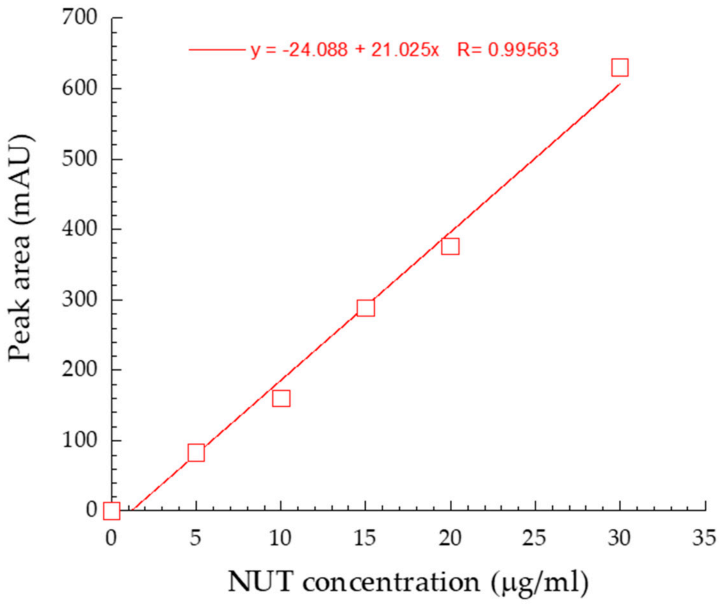

Column	Mobile Phase	Wavelength	Flowrate	Elution Time
C-18 reverse-phase column (15 × 0.46 cm)	acetonitrile/water 40:60 v/v, pH 3	255 nm	1 mL/min	9 min

Figure S2. HPLC calibration curve of standard NUT obtained by the reported conditions.

Table S1. Zeta potential values of the indicated vesicular systems.

	ETO	ETO-NUT$_{0.3}$	T-ETO	T-ETO-NUT$_{0.3}$
**Z-potential**	−16.2 ± 4.5	−25.5 ± 6.8	−20.4 ± 7.7	−28.8 ± 4.3

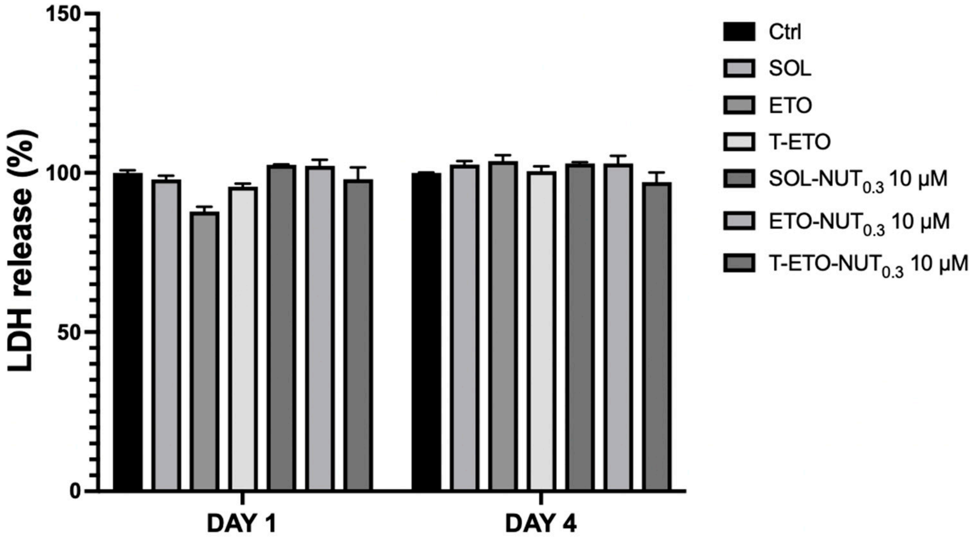

Figure S3. LDH assay on skin biopsies treated with SOL, ETO and T-ETO unloaded or loaded with NUT$_{0.3}$ at the dose of 10 μM for 1 day (DAY 1) up to 4 days (DAY4). 10 μM was selected as the treatment dose to use for the experiment based on previous studies conducted on primary keratinocytes. Lactate dehydrogenase released levels were measured in media of skin explants and normalized to the negative control (Ctrl), considered as the 100% of skin explants viability. Data are the average ± SD of three independent experiments.
